# Ostéoporose endocrinienne: à propos d'une série de cas

**DOI:** 10.11604/pamj.2015.22.358.7306

**Published:** 2015-12-11

**Authors:** Naima Bouznad, Khadija Diyane, Ghizlane El Mghari, Ahlam Belkhou, Nawal El Ansari

**Affiliations:** 1 Service d'Endocrinologie, Diabétologie et Maladies Métaboliques, Laboratoire PCIM, FMPM, CHU Mohamed VI, Marrakech, Maroc; 2 Service de Rhumatologie, CHU Mohamed VI, Marrakech, Maroc

**Keywords:** Ostéoporose, ostéopénie, hypogonadisme, hypercorticisme, Osteoporosis, osteopenia, hypogonadism, hypercortisolism

## Abstract

L'ostéoporose endocrinienne devrait devenir rare aujourd'hui puisque les endocrinopathies ont bénéficié d'un diagnostic plus précoce. Cependant elle reste fréquente, sous diagnostiquée et peu prise en charge. Sa gravité est essentiellement liée au risque fracturaire, et au risque élevé de morbi-mortalité. Le but de notre travail est de déterminer le profil ostéodensitométrique des patients suivis pour endocrinopathie et de définir les caractéristiques de l'ostéoporose et de l'ostéopénie chez ces patients. Il s'agit d'une étude de type transversale, descriptive portant sur 63 patients suivis pour endocrinopathies au service d'Endocrinologie-Diabétologie du CHU Mohamed VI de Marrakech, s’étalant sur une période de 02 ans allant du début du mois de Janvier 2012 au mois de Janvier 2014. La moyenne d’âge des patients était de 36,30 ± 15,38 ans, avec un sex-ratio de 0,31. Les pathologies endocriniennes étaient dominées par l'hypogonadisme (32%), et par l'hypercorticisme (19%), suivis par l'hyperthyroïdie (11%). Le T-score moyen au niveau du rachis et au niveau du fémur était respectivement de -1,65% ± 1,88 DS, et -0,85 ± 1,70 DS. L'atteinte de la densité minérale osseuse a été plus fréquente au niveau du rachis (61,9%). L'ostéoporose a été surtout constatée au cours des hypogonadismes, hypercorticismes, hyperthyroïdies et hyperparathyroïdies. Néanmoins elle a été observée également au cours des hypothyroïdies et d'autres étiologies non connues pour être responsables d'une ostéoporose. Ce travail nous a ainsi conduits à situer l'intérêt de rechercher systématiquement l'atteinte osseuse devant toute endocrinopathie susceptible de provoquer une atteinte rhumatologique.

## Introduction

La plupart des pathologies endocriniennes ont un retentissement osseux qui est pris en compte dans la morbidité de ces maladies. En effet, le tissu osseux fait l'objet d'un perpétuel remodelage sous contrôle, entre autres, de facteurs hormonaux. Ainsi, la plupart des désordres endocriniens s'accompagnent de remaniements de l'appareil ostéo-articulaire. Il s'agit principalement de l'ostéoporose [1]. Cette dernière est définie comme étant une maladie générale du squelette caractérisée par une diminution de la résistance osseuse prédisposant le patient à un risque accru de fractures [2]. L'ostéoporose endocrinienne, qui devrait devenir rare aujourd'hui en raison d'un traitement efficace instauré précocement, évolue parfois à bas bruit et peut être la première en date. C'est surtout le cas pour l'hyperparathyroïdie primitive [1]. L'ostéodensitométrie (ODM) est un outil pour l’évaluation du retentissement osseux des endocrinopathies, mais aussi un bon marqueur de sévérité clinique et sécrétoire de cette endocrinopathie. La gravité de l'ostéoporose réside dans le risque accru de fracture, ayant des conséquences importantes en termes de mortalité et de morbidité. Pour cela, la prévention des fractures dues à l'ostéoporose a été déclarée « cause prioritaire » par l'OMS en 2000 [2]. Le but de notre travail est de déterminer le profil ostéodensitométrique des patients suivis pour endocrinopathie et de définir les caractéristiques de l'ostéoporose et de l'ostéopénie chez ces patients, mais aussi de mettre l'accent sur la nécessité d'une prise en charge adéquate de cette pathologie souvent négligée.

## Méthodes

Il s'agit d'une étude transversale, à visée descriptive étendue sur une période de 02 ans allant du début du mois de Janvier 2012 au mois de Janvier 2014, intéressant les patients suivis pour endocrinopathie au service et en consultation d'Endocrinologie-Diabétologie du CHU Mohamed VI de Marrakech. Les critères d'exclusion sont: les patients suivis pour un rhumatisme inflammatoire et les patients suivis pour une maladie systémique. La saisie et l'analyse des données ont été réalisées à l'aide du logiciel SPSS version 10.

## Résultats

**Caractéristiques démographiques et cliniques**: La moyenne d’âge de nos patients était de 36,30 ± 15,38 ans, avec des extrêmes de 12 à 73 ans. On note une nette prédominance du sexe féminin, avec un Sex-ratio de 0,3. La moyenne de l'indice de masse corporelle des patientes était de 25,12 ± 6,22 Kg/m^2^. Tandis que celle des patients était de 23,72 ± 7,28 Kg/m^2^. Les femmes ménopausées représentaient 27,1% de la population de l’étude. Les autres comorbidités sont représentées par l'hypertension artérielle (20,6%), le diabète (14,3%), la dyslipidémie (7,9%) et le tabagisme (4,8%).

**Figure 1:**
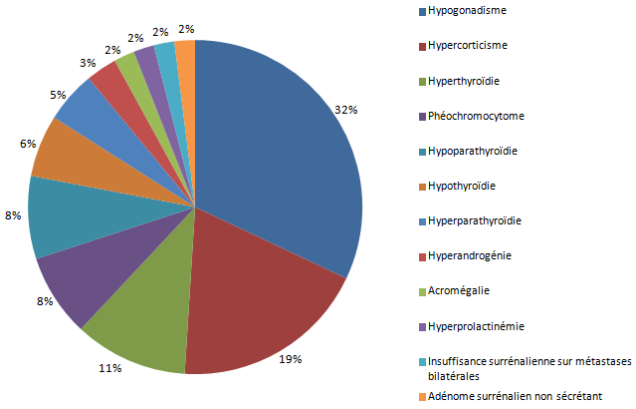
Nature de l'endocrinopathie

**Nature de l'endocrinopathie**: le motif de consultation des patients était dominé par l'aménorrhée primaire et secondaire (16%), le syndrome de cushing (16%) et les hyperthyroïdies (10%). Le délai entre le début des symptômes et la première consultation était de 5,5 ans ± 8,3 ans. Les pathologies endocriniennes chez cette population étaient très diversifiées. Elles étaient dominées par l'hypogonadisme rencontré dans 32% des cas et par l'hypercorticisme dans 19% des cas, suivis par l'hyperthyroïdie constatée chez 11% des patients (Figure 1).

**Figure 2:**
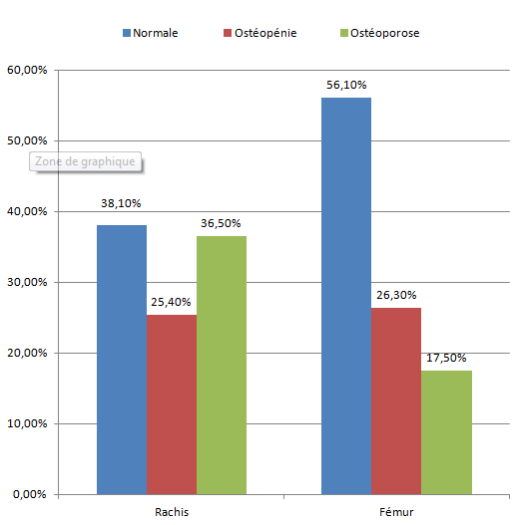
Fréquence de l'atteinte de la densité minérale osseuse

**Fréquence de l'atteinte osseuse selon la localisation**: concernant la densité minérale osseuse (DMO), le T-score moyen au niveau du rachis était de -1,65% ± 1,88 DS le qualifiant d'ostéopénique, avec un T-score moyen fémoral de -0,85 ± 1,70 DS. L'atteinte de la DMO a été plus fréquente au niveau du rachis (61,9%) avec une ostéoporose dans 36,5% des cas et une ostéopénie dans 25,4% des cas. L'atteinte fémorale a été surtout une ostéopénie, présente chez 26,4% des patients avec une ostéoporose chez seulement 17,5% des cas (Figure 2).

**Corrélation entre la DMO et le siège de l'atteinte osseuse: rachis/ fémur**: l'analyse statistique avait montré qu'Il y a une relation significative entre la DMO et le siège de l'atteinte osseuse. L’étude de corrélation entre le T-score du rachis et le T-score du fémur avait montré qu'il y a une relation linéaire entre ces deux variables: Plus la valeur de la DMO du rachis diminue, plus celle de Fémur diminue aussi (coefficient de corrélation = 0,811) (Tableau 1).

**Tableau 1:** Corrélation entre la de la densité minérale osseuse et le siège de l'atteinte osseuse

			ODM			Total
			Normale	Ostéopénie	Ostéoporoe	
Siège	Fémur	Effectif	32	15	10	57
		ODM	57,1%	48,4%	30,3%	47,5%
	Rachis	Effectif	24	16	23	63
		ODM	42,9%	51,6%	69,7%	52,5%
Total		Effectif	56	31	33	120
		ODM	100,0%	100,0%	100,0%	100,0%

### Fréquence de l'atteinte osseuse selon le type de l'endocrinopathie

**Au cours de l'hypogonadisme**: les anomalies de la DMO rachidienne ont été constatées dans 75% des cas avec hypogonadisme, avec essentiellement une ostéoporose dans 45% des cas. Cette dernière avait concernée 13,3% des cas au niveau du fémur, avec une ostéopénie fémorale dans 40% des cas.

**Au cours de l'hypercorticisme:** chez les patients avec hypercorticisme, l'atteinte de la DMO rachidienne avait concerné 66,7% des patients, avec une ostéoporose dans 50% des cas et une ostéopénie dans 16,7% des cas. L'atteinte de la DMO fémorale a été constatée chez 33,3% des patients.

**Au cours de l'hyperthyroïdie**: le pourcentage de l'ostéoporose rachidienne a été de 33,3% au cours de l'hyperthyroïdie, tandis que l'ostéopénie rachidienne a été observée chez 33,3% des patients. Au niveau du fémur, l'ostéopénie a été constatés chez 66,7% des patients.

**Au cours de l'hyperparathyroïdie**: parmi les patients ayant une hyperparathyroïdie, 33,3% avaient une ostéoporose rachidienne, et 33,3% avaient une ostéopénie rachidienne. L'atteinte de la DMO fémorale a été constatée chez 66,7% des patients.

**Au cours des autres endocrinopathies**: l'analyse de l'atteinte de la DMO dans les autres endocrinopathies avait montré une ostéoporose dans 25% des cas d'hypothyroïdie, dans 50% des cas phéochromocytome, et dans 22,2% des cas d'hyperparathyroïdie. L'ostéopénie a était constatée respectivement chez 37,5%, 25% et 22,2% des patients.

**Figure 3:**
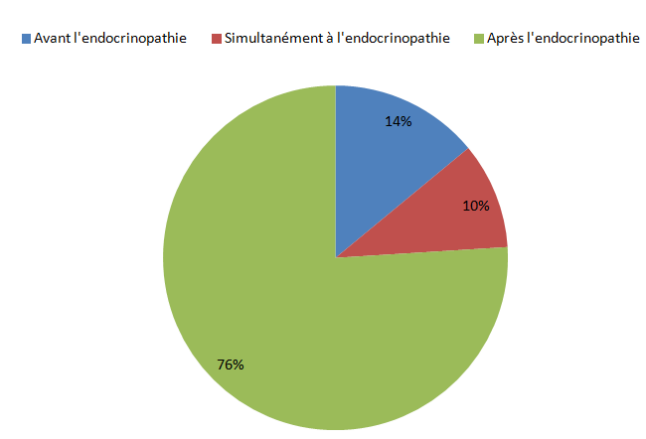
Chronologie d'apparition de l'atteinte de la densité minérale osseuse

**La chronologie d´apparition de l'atteinte de la DMO**: l'atteinte de la DMO a été diagnostiquée essentiellement après ou simultanément à la découverte de l'endocrinopathie, par contre elle en était révélatrice dans 14% des cas (Figure 3).

## Discussion

La plupart des endocrinopathies peuvent s'accompagner de complications ostéoarticulaires notamment d'une ostéoporose. En effet La plupart des glandes endocrines, via les hormones qu'elles sécrètent, peuvent influencer la balance formation-résorption, le plus souvent au profit de la résorption. Le tissu osseux fait l'objet d'un perpétuel remodelage lié schématiquement à l'action conjuguée des ostéoclastes (résorption) et des ostéoblastes (formation). Cette balance formation-résorption est placée sous l'influence de nombreux facteurs, notamment nutritifs et endocriniens [1]. De nombreuses hormones sont impliquées dans la régulation du remodelage osseux.

### Les facteurs influençant le remodelage osseux

#### Facteurs d'origine endocrinienne

Certaines ont un effet anabolique sur le tissu osseux en favorisant l'action des ostéoblastes ou en inhibant celle des ostéoclastes: l'hormone de croissance (GH) et l'insulin-like growth factor 1 (IGF1), l'insuline et les stéroïdes sexuels (androgènes surrénaliens et gonadiques, et estrogènes) [3]. D'autres hormones, au contraire, favorisent la résorption osseuse: les glucocorticoïdes, la parathormone, et les hormones thyroïdiennes (T3, T4).

#### Facteurs d'origine locale

Il s'agit des prostaglandines dont la PGE2, des interleukines, de facteurs de croissance épidermiques qui sont des acteurs locaux activateurs des ostéoclastes. À l'inverse, le monoxyde d'azote (NO) inhibe la résorption osseuse [4].

#### Contraintes mécaniques

L'activité physique a également un effet sur le remodelage osseux. Les forces de déformation et de pression modifient l'activité de remodelage osseux. Une ostéopénie est d'ailleurs observée après un séjour en microgravité [4].

### Ostéoporose au cours des hypercorticismes

L'ostéoporose cortisonique est la première cause des ostéoporoses secondaires. Longtemps négligée, son importance grandit, notamment dans le cadre de nouvelles indications de corticothérapie au long cours. En effet Il existe un lien épidémiologique certain entre la corticothérapie et les fractures. Certaines études estiment même que des fractures surviennent chez 30 à 50% des patients sous corticoïdes au long cours. Enfin, l'excès de corticoïdes favorise l'apparition d'un hypogonadisme et la diminution de production d'androgènes surrénaliens [5]. Les mécanismes et les conséquences de l'atteinte osseuse liée aux glucocorticoïdes (GC) sont mieux compris, mais l'ostéoporose cortisonique reste sous-diagnostiquée et sa prise en charge insuffisante. L'hypercorticisme chronique (ou syndrome de Cushing) est responsable d'une augmentation du catabolisme osseux, du fait d'une activation des ostéoclastes, d'une inhibition de l'activité ostéoblastique et d'une inhibition de l'absorption intestinale du calcium. L'hypercorticisme peut être iatrogène ou endogène. L'ostéoporose est principalement visible dans les régions où l'os trabéculaire prédomine (extrémités supérieures des fémurs, côtes et rachis). La densité minérale osseuse est diminuée et le risque de fracture est augmenté, en particulier au rachis lombaire où cette complication survient chez 70% des patients. Les fractures vertébrales sont souvent asymptomatiques. Dans notre série, l'atteinte de la DMO rachidienne avait concerné 66,7% des patients, ce qui rejoint les données de la littérature. Il s'agissait essentiellement d'une ostéoporose dans 50% des cas et d'une ostéopénie dans 16,7% des cas. L'atteinte de la DMO fémorale a été également constatée chez 33,3% des patients.

### Ostéoporose au cours des hypogonadismes

L'hypogonadisme est la deuxième cause d'ostéoporose secondaire après la corticothérapie [6]. Il représente environ 20 à 30% des ostéoporoses secondaires de l'adulte. La régulation de la sécrétion des hormones sexuelles est sous le contrôle de l'axe hypothalamo-hypophysaire influençant la sécrétion hypophysaire des gonadotrophines: folliculo-stimulating hormone (FSH) et hormone lutéinisante (LH). Celles-ci, libérées agissent ensuite sur les gonades pour stimuler la sécrétion des hormones sexuelles (estrogènes et progestérone chez la femme ou testostérone chez l'homme) qui ont un effet anabolique sur le tissu osseux, alors que la FSH et LH favorisent un remodelage osseux. La dysfonction des glandes endocrines sexuelles correspond à un hypogonadisme, c'est-à-dire à un déficit d'hormones sexuelles ayant des conséquences importantes sur la DMO. La carence oestrogénique s'accompagne d'un hyper-remodelage osseux lié à une augmentation de l'activité ostéoclastique. L'excès de FSH observé au cours des hypogonadismes d'origine ovarienne, par levée du rétrocontrôle négatif, expliquerait également en partie la perte osseuse via une stimulation directe de l'activité ostéoclastique. Les étiologies sont multiples. On distingue les étiologies supra-hypothalamiques, les étiologies hypothalamo-hypophysaires et les étiologies périphériques [6]. Chez la femme, le degré de perte osseuse est variable en fonction de l'intensité et de la durée de l'hypoestrogénie: perte de 5 à 8% par an, encore plus importante chez la femme jeune [7]. Dans notre série, Les anomalies de la DMO du rachis étaient plus fréquentes au cours de l'hypogonadisme que dans l'hypercorticisme. En effet elles étaient observées chez 75% des patients, avec essentiellement une ostéoporose dans 45% des cas. L'atteinte fémorale était également fréquente. Elle avait concernée 53,3% des patients, avec essentiellement une ostéopénie (40%). Cette fréquence élevée est peut être liée à un biais de sélection, puisque l'hypogonadisme avait concerné plus des deux tiers de notre population.

### Ostéoporose au cours des hyperparathyroïdies

La PTH stimule la résorption osseuse et donc le flux de calcium et de phosphate de l'os vers le plasma. Elle augmente la réabsorption tubulaire du calcium et diminue celle des phosphates. Elle stimule enfin la 1’-hydroxylase rénale dans le tubule proximal. L'hypersécrétion de PTH par les glandes parathyroïdes est la conséquence d'une production excessive, inappropriée de PTH ayant pour principale conséquence métabolique une hypercalcémie. La plupart des hyperparathyroïdies sont asymptomatiques, de découverte fortuite au cours d'un bilan biologique ou d'une échographie cervicale [8]. L'hyperparathyroïdie est généralement responsable d'une ostéoporose secondaire par augmentation de la résorption osseuse. L'atteinte prédomine sur l'os cortical, et à un moindre degré à l'extrémité supérieure du fémur. Le pourcentage de patients porteurs d'une hyperparathyroïdie primitive ayant une DMO inférieure à -0,8 DS en Z-score est seulement de 13% pour le rachis lombaire et de 23% pour le fémur. Néanmoins, il a été mis en évidence un sous-groupe de patients atteints d'hyperparathyroïdie primitive avec atteinte rachidienne prédominante qui représente environ 15% à 20% des hyperparathyroïdies primitives [9]. Les résultats de notre étude avaient par contre montré que la fréquence de l'atteinte de la DMO rachidienne était fréquente et égale à celle du fémur: 66,7% des patients ayant une hyperparathyroïdie. Elle était même plus grave au niveau du rachis, avec une ostéoporose rachidienne dans 33,3% des cas. Ceci pourrait être expliqué par la longue durée d’évolution, mais aussi par le faible effectif du sous-groupe d'hyperparathyroïdie.

### Ostéoporose au cours des hyperthyroïdies

Les hormones thyroïdiennes (T3, T4) sont des activateurs du métabolisme de base. Leur sécrétion est placée sous le contrôle de la TSH hypophysaire, elle même placée sous l'influence de la sécrétion hypothalamique de TRH. Chez l'enfant, elles jouent principalement un rôle dans la croissance et la maturation du tissu osseux [1]. L'ostéoporose thyroïdienne est une ostéopathie cortico-trabéculaire due à une accélération du remodelage osseux par les effets directs des hormones thyroïdiennes sur le tissu osseux [10]. La densitométrie est 10 à 20% plus basse chez les hyperthyroïdiens par rapport à des sujets de même âge et de même sexe. La prévalence des fractures est mal appréciée dans la littérature [10]. L'ostéoporose thyroïdienne peut se rencontrer au cours de toutes les causes d'hyperthyroïdie. Le traitement de l'hyperthyroïdie, quelle que soit son origine, endogène ou exogène, entraîne une correction rapide de l'excès du remodelage et une récupération osseuse progressive [3]. Dans notre étude, l'atteinte de la DMO rachidienne et fémorale au cours de l'hyperthyroïdie était présente chez 66,7% respectivement. L'ostéoporose a été constatée seulement au niveau du rachis, et ce chez 33,3% des patients; ceci peut être expliquer le faite que la plupart des hyperthyroïdies sont suivies en consultation, hors notre étude est réalisée chez les patients en milieu hospitalier.

### Ostéoporose au cours des autres endocrinopathies

Les autres endocrinopathies étaient très peu représentées dans notre série avec un effectif insuffisant pour pouvoir en déduire des conclusions.

## Conclusion

L'atteinte de la DMO au cours des pathologies endocriniennes est fréquente mais reste sous diagnostiquée et peu prise en charge. Sa gravité est liée au risque accru de complications, entre autres une augmentation du risque fracturaire, qui est un puissant facteur de risque de morbi-mortalité prouvé dans la littérature. La grande fréquence en population générale et l'importance du retentissement osseux des hyperparathyroïdies, hyperthyroïdies et des situations d'hypogonadisme et d'hypercorticisme les placent parmi les pathologies endocriniennes pour lesquelles l’évaluation osseuse est la mieux connue. Ce travail montre une fréquence élevée de l'ostéoporose et de l'ostéopénie chez les patients suivis pour des pathologies endocriniennes, même les moins connues pour l'avoir entrainée. Il nous a ainsi conduits à situer l'intérêt de rechercher systématiquement l'atteinte osseuse devant toute endocrinopathie susceptible de provoquer une atteinte rhumatologique. Mais aussi l'intérêt d'un bilan étiologique bien conduit, à la recherche d'une pathologie endocrinienne devant toute ostéoporose ou ostéopénie.
